# Modulation of Neural Activity during Guided Viewing of Visual Art

**DOI:** 10.3389/fnhum.2017.00581

**Published:** 2017-11-30

**Authors:** Guillermo Herrera-Arcos, Jesús Tamez-Duque, Elsa Y. Acosta-De-Anda, Kevin Kwan-Loo, Mayra de-Alba, Ulises Tamez-Duque, Jose L. Contreras-Vidal, Rogelio Soto

**Affiliations:** ^1^Tecnológico de Monterrey, National Robotics Laboratory, School of Engineering and Sciences, Monterrey, Mexico; ^2^INDI Ingeniería y Diseño S.A.P.I. de C.V., Monterrey, Mexico; ^3^Laboratory for Non-invasive Brain-Machine Interface Systems, Department of Electrical and Computer Engineering, University of Houston, Houston, TX, United States

**Keywords:** neuroaesthetics, EEG, non-laboratory, MoBI, BCI, aesthetics, art, beta band

## Abstract

Mobile Brain-Body Imaging (MoBI) technology was deployed to record multi-modal data from 209 participants to examine the brain’s response to artistic stimuli at the Museo de Arte Contemporáneo (MARCO) in Monterrey, México. EEG signals were recorded as the subjects walked through the exhibit in guided groups of 6–8 people. Moreover, guided groups were either provided with an explanation of each art piece (Guided-E), or given no explanation (Guided-NE). The study was performed using portable Muse (InteraXon, Inc, Toronto, ON, Canada) headbands with four dry electrodes located at AF7, AF8, TP9, and TP10. Each participant performed a baseline (BL) control condition devoid of artistic stimuli and selected his/her favorite piece of art (FP) during the guided tour. In this study, we report data related to participants’ demographic information and aesthetic preference as well as effects of art viewing on neural activity (EEG) in a select subgroup of 18–30 year-old subjects (Nc = 25) that generated high-quality EEG signals, on both BL and FP conditions. Dependencies on gender, sensor placement, and presence or absence of art explanation were also analyzed. After denoising, clustering of spectral EEG models was used to identify neural patterns associated with BL and FP conditions. Results indicate statistically significant suppression of beta band frequencies (15–25 Hz) in the prefrontal electrodes (AF7 and AF8) during appreciation of subjects’ favorite painting, compared to the BL condition, which was significantly different from EEG responses to non-favorite paintings (NFP). No significant differences in brain activity in relation to the presence or absence of explanation during exhibit tours were found. Moreover, a frontal to posterior asymmetry in neural activity was observed, for both BL and FP conditions. These findings provide new information about frequency-related effects of preferred art viewing in brain activity, and support the view that art appreciation is independent of the artists’ intent or original interpretation and related to the individual message that viewers themselves provide to each piece.

## Introduction

Mobile Brain-Body Imaging (MoBI) systems have been of interest in the field of psychophysiology for almost 10 years (Makeig et al., 2009) and have recently become increasingly popular as tools for neuroscience research and therapy (Wagner et al., 2012; Ordikhani-Seyedlar et al., 2016), as well as entertainment devices (Abdulkader et al., 2015; Speier et al., 2016), allowing quantification and visualization of human neurophysiological and behavioral elements otherwise only subjectively expressed, including human cognitive functions, emotions, motor intentions and human–human interaction dynamics in varied circumstances. Recently, efforts have focused on increasing knowledge about emotion-induced brain wave changes (Makeig et al., 2011), as well as on evaluating MoBI’s ability to successfully identify human emotional changes (Oude Bos, 2006; Schaaff and Schultz, 2009; Liu et al., 2011; Rached and Perkusich, 2013). A few studies have also specifically explored brainwave pattern changes during artistic stimulation, either by acquiring data from art performers or from spectators as study subjects (Shourie et al., 2014; Kontson et al., 2015), in an effort to study the neural basis of the aesthetic perception and of the emergence of creativity in natural complex settings (Picard et al., 2001; Oude Bos, 2006; Cruz-Garza et al., 2016). Research in this area has shown subjects exposed to art provide excellent opportunities to identify relationships between humans’ inner brain states and contextual exposures to artistic aesthetics (Fetz, 2012; Aviv, 2014; Kontson et al., 2015), also leading to results showing that appreciation of visual arts is influenced by an art piece’s popularity, lexical characteristics of the title and description, the specific type of art to which subjects are exposed (e.g., figurative versus abstract), and the amount of time exposed to art pieces (Brieber et al., 2014, Mastandrea and Umiltà, 2016; Ordikhani-Seyedlar et al., 2016). Neuroaesthetic studies have additionally started to explore the neural effects of engagement in artistic activities (Leslie et al., 2014a) and shown that although providing limited information to subjects during the appreciation of an art piece (i.e., author, technique, original title of painting) may lead to increased understanding of its message, it does not influence changes in neural emotional response or art preference (Aviv, 2014; Mastandrea and Umiltà, 2016). However, in regards to achieving such engagement in musical activities via hand motions, frequency-specific effects have indeed been observed (Leslie et al., 2014b).

Studies based on different neuroimaging strategies, such as functional magnetic resonance imaging (fMRI), have also shed light on neural activity correlation with aesthetic experiences, showing subjects experiencing representational art activate certain areas of the brain which correlate with emotion, interpretation and perception (Huang, 2009; Brown et al., 2011; Vessel et al., 2013; Brieber et al., 2014; Beaty et al., 2016). Conversely, studies have also shown abstract art to be linked with a general brain activation instead of with area-specific events due to subjects’ inability to uncover or create object-relations while being stimulated by this art style (Aviv, 2014).

Although much has been learned through the aforementioned studies, it is still generally agreed that a laboratory setting presents limitations in the true analysis of actual brain responses, primarily because these anti-naturalistic settings often resort to digital representations of paintings instead of using actual pieces, and because of movement constraints that eliminate a large part of neural activity related to motion intentions (Brieber et al., 2014; Mastandrea and Umiltà, 2016). Recording EEG signals while allowing free motion of subjects presents a challenge because of the ease with which artifacts may be generated by such unconstrained motions; however, recent advances in analysis methods (Gwin et al., 2010; Bono et al., 2016; Hashemi et al., 2016 and those presented herein) have presented new alternatives to effectively account for these artifacts, using mathematical and data-driven strategies. Furthermore, non-laboratory experiments are crucial in integrating a more complete understanding of neural activity related to the actual daily aesthetic experience and how it may affect brain function and even counter chronic neural conditions.

Fortunately, and despite still being in their early stages of utilization and development, modern and portable MoBIs technologies offer important opportunities to examine neural activity ‘in action and in context’ allowing researchers to take experiments out of the laboratory and into complex natural settings such as museums.

In this study, we aim to build upon the findings of the above studies as well as to address the limitations regarding experiments performed in laboratory settings by exploiting the advantages of portable MoBIs. The study was carried out with a large population of visitors with rich demographics during guided and non-guided tours of the temporary, expressionist art exhibit by Otto Dix at the Museo de Arte Contemporáneo (MARCO) in Monterrey, México. A cohort of subjects was selected based on age (18 – 30 years old) for analysis of brain activity during exhibit tours and with relation to variables including gender and sensor placement as well as presence or absence of artistic explanation. We report data related to participants’ demographic information and aesthetic preference as well as characteristics of brain activity in relation to the aforementioned variables as well as to relaxation and artistic stimulation states.

## Materials and Methods

An observational-analytical protocol was developed to study the brain response of visitors at MARCO over a period of 2 months. The study was conducted according to the Declaration of Helsinki and approved by an Independent Ethics Committee at the Instituto Tecnológico de Monterrey, Monterrey, Mexico.

### Subject Preparation

**FIGURE 1:**
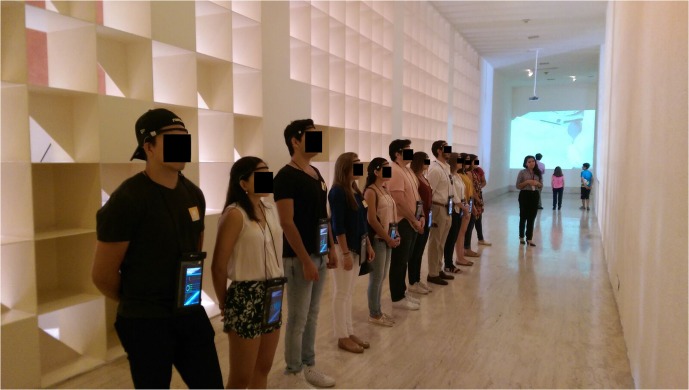
Baseline (BL) recording. Subjects were asked to stare at a white wall with eyes open, to clear their minds and to remain motionless for 60 s. BL recordings were performed on all subjects before each tour through an art exhibit started.

Upon informed consent approval to participate in the study, subjects were fitted with the portable non-invasive brain-body imaging (MoBI) technology. Participants were also asked to stare at a white wall during 1 min before any artistic stimulation, in order to induce peacefulness and generate a neural-data baseline (BL) reference which was influenced by no aesthetic or motion-related stimulation, as seen in **Figure 1**. This recording was performed on all subjects before each tour started.

### Data Acquisition

**FIGURE 2:**
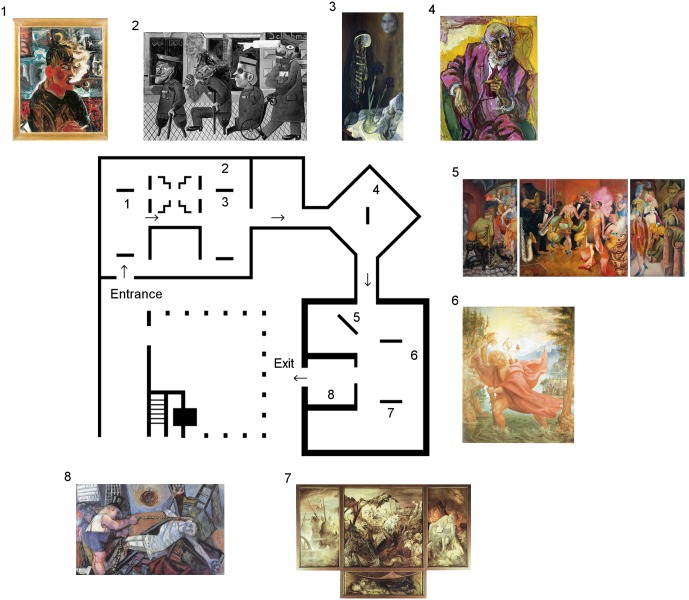
Museum’s map of expressionist art exhibit by Otto Dix within MARCO. Art pieces included in experimental tours are numbered and shown in their actual positions within the exhibit room. All participants in different groups were led through the exhibit in the same incremental order with relation to art piece numbering, beginning with art piece 1 and finishing with art piece 8. *(Images were extracted from public sites. 1: http://www.march.es/arte/madrid/exposiciones/dix/obras/autorretratof.asp; 2: http://www.ottodix.org/catalog-item/129.006/; 3, 6, 7: http://www.all-art.org/art_20th_century/dix1.html; 4: http://www.gemäldekopien-aigner.de/index.php?wahl=G1&bname=4_2; 5: https://www.facinghistory.org/resource-library/image/otto-dix-gross-stadt-metropolis-1928; 8: https://www.pinterest.co.uk/pin/376824693807329152/).*

Demographic and behavioral data was acquired during the guided tours through the exhibit shown in **Figure 2**. Subjects walked in guided groups of 6–8 people during viewings with instructions of avoiding talking to each other. Nevertheless, the experimenters logged instances where participants talked to each other for consideration in data segmentation and selection. These data (and that of subjects who continuously spoke, showed uncommonly large head motions, or who were chewing gum) were considered artifactual and not used in the analysis. Time was tracked with a chronometer in order to identify permanence of participants in specific art pieces; this was also useful for later coordination of EEG data with aesthetic references, leading to algorithmic data interpretation. A written log of subjects activity and general behavioral state was also kept and matched to measured times, aiding in the process of artifact removal; additionally, participants were also given a pocket digital-video camcorder (Conbrov, ShenZhen, China), which recorded each subject’s track over the exhibit and provided additional information for activity tracking. At the end of the tour, subjects were asked to complete a questionnaire where data relating to their occupational status, their level and frequency of art consumption, and their aesthetic preference (favorite painting within the exhibit) were recorded.

EEG data acquisition was performed using 4-electrode, non-invasive portable Muse headbands (InteraXon Inc. Toronto, Ontario M5V1K4, Canada) worn by subjects during the entire experimental process. Electrodes in the headband were located on the prefrontal and temporo-parietal lobes, including locations TP9 (S1), AF7 (S2), AF8 (S3), and TP10 (S4), in accordance to the international 10–20 EEG-electrode positioning system. Data was first acquired through Muse-io (InteraXon Inc. Toronto, Ontario M5V1K4, Canada), saved by MuseSaver (InteraXon Inc. Toronto, ON M5V1K4, Canada), and displayed in real time with MuseTimeTracerV2 (InteraXon Inc. Toronto, Ontario M5V1K4, Canada). All three software were run in real-time over an HP mini-tablet (HP Inc. Palo Alto, CA 94304, United States) while subjects experienced the exhibit. Data was later downloaded in one Windows PC using a modified version of Muse Software Developer’s Kit (InteraXon Inc. Toronto, Ontario M5V1K4, Canada) and processed offline using MATLAB software (MathWorks, Natick, MA 01760, United States) for data analyses. Additionally, the device’s internal accelerometer consists of three-axis inertial units sampling at 50 Hz, with 10 bit resolution, and with a range of ±2 [G]. The external camcorder recorded 720P HD video on a 75° wide-angle lens.

### Data Analysis: Preprocessing and Denoising

**FIGURE 3:**
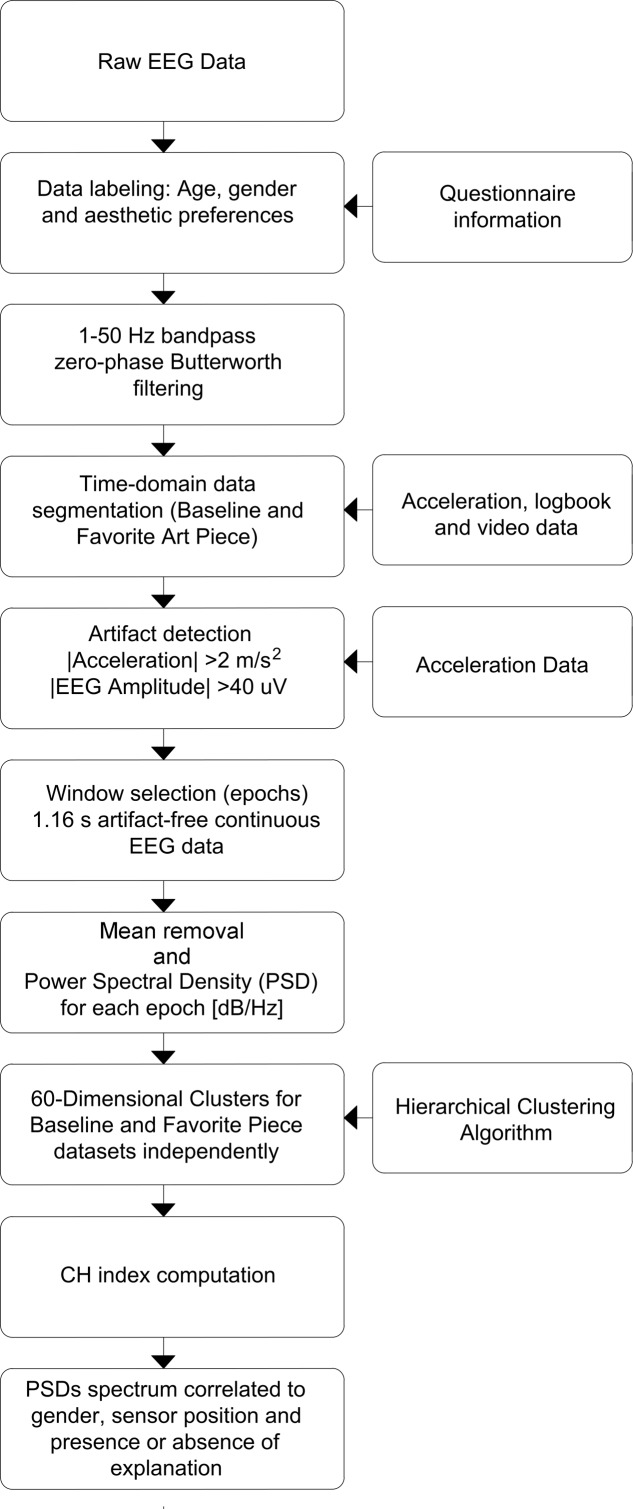
Data processing flowchart. Preprocessing, denoising and processing steps are shown in sequence. Preprocessing focuses on time-domain manipulation of data, while processing was performed on data in frequency domain.

Raw EEG data was first labeled using information from anonymous questionnaires, that include information about age, gender, aesthetic preference and presence or absence of explanation, linked to the EEG headsets. Signal filtering was performed using a zero-phase Butterworth bandpass filter for frequencies 1–50 Hz. Information from the experiment’s logbook, headset’s acceleration data and video data were used to identify and divide EEG signals into segments corresponding to readings for baseline and favorite painting. To identify usable (e.g., artifact-free) data segments corresponding to each specific painting and to baseline state, we used a multipronged approach: first, logbook indications were used to identify if there was a specific reason for individual subjects not to be considered in the analysis (e.g., because they were chewing gum); second, times on logbooks were used to identify subject locations within the exhibit (times were logged for groups and notes were made if an individual subject did not follow his group’s patterns, for any reason) and video recordings were used for confirmation of log-book notes; third, accelerometer data and EEG amplitude changes were used to identify artifactual periods of time (segments of time with absolute accelerations > 2 [m/s2] on all three axes and EEG amplitudes over 40 [μV] were deemed artifactual); fourth, location-labeled data which was not identified as artifactual were segmented into epochs which were used for the analysis. Windows of 256 samples, or 1.16 [s] were selected from the middle of the viewing periods free of artifacts, resulting in 1,531 epochs for BL data set and 1,312 epochs for FP data set. The process is shown in **Figure 3**.

### Data Analysis

Power Spectral Density (PSD) was chosen the primary variable of interest: one PSD was generated per epoch (256 samples; 1.16 [s]) using Matlab’s Thomson’s Multitaper Power Spectral Density Estimate function *(‘pmtm’; sampling frequency: 220 Hz)*. 60-Dimensional vectors comprising the baseline-corrected power spectral density were separated into clusters generated using Hierarchical Clustering using the Matlab’s Machine Learning and Statistics Toolbox (‘clustergram’; linkage method: ward, inner squared distances using Euclidian distance; standardized columns) as in Yu et al., 2016; Ieracitano et al., 2017. The purpose was to identify the patterns of spectral content and their spectral variability across conditions and to examine potential gender differences. *N* data points were distributed in K clusters using 2 alternative cluster segmentation approaches to note similarities and differences of PSDs correlated to gender, sensor position, and presence or absence of explanation of the art pieces viewed: *Kn1* number of clusters were chosen according to CH index (defined in the next paragraph) and *Kn2* clusters following a *K* = number of variables + 1 strategy. The complete preprocessing and processing procedure is described in **Figure 3**.

*CH index* was used to quantify adequateness of using k number of clusters to divide *n* number of data points. The greater the index, the more adequately does a specific number of clusters divide a specific dataset. The index considers *W* (separation of points within independent clusters) and *B* (separation of independent clusters from each other) to determine clustering adequateness. Equations 1–5 describe the calculation process of the index.

$$CH(k)=\frac{B(K)/\left(k-1\right)}{w(K)/\left(n-K\right)}$$

$$W=\overset{K}{\underset{k=1}{\Sigma }}\underset{c(i)=k}{\Sigma }{\left||X_{i}-\overset{¯}{X}|\right|}_{2}^{2}$$

$$B=\overset{K}{\underset{k=1}{\Sigma }}n_{k}{\left||{\overset{¯}{X}}_{k}-\overset{¯}{X}|\right|}_{2}^{2}$$

$${\overset{¯}{X}}_{k}=\frac{1}{n_{k}}\underset{C(i)=k}{\Sigma }X_{i}$$

$$\overset{¯}{X}=\frac{1}{n}\overset{n}{\underset{i=1}{\Sigma }}X_{i}$$

For both baseline and favorite painting data sets, *CH index* indicated two clusters presented the best segmentation distances for the respective data points.

## Results

### Demographics, Artistic Preference and Subject Behavior

#### Demographics

**FIGURE 4:**
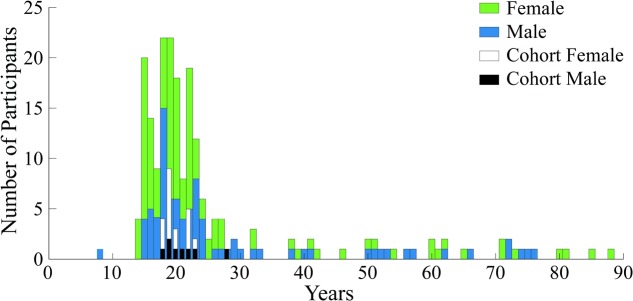
Demographics histogram for all participants and the analysis cohort. Age and gender are shown for the *N* = 209 subjects who experienced the expressionist art exhibit by Otto Dix. Age and gender are also shown for the analysis cohort.

Voluntary participants (*N* = 209) in the study were between the ages of 6 and 88 years old; with 86 (41%) male and 123 (59%) female. **Figure 4** shows the gender and age distribution for the participants. Moreover, **Figure 4** also shows an additional cohort comprised of a portion of young adult subjects between the ages of 18 and 30, from the entire pool, which was used for the second part of our analyses to eliminate the age confound. The rationale and selection process for this second group is detailed in section “Neural Activity Related to Aesthetic Preference in Young Adults.”

**FIGURE 5:**
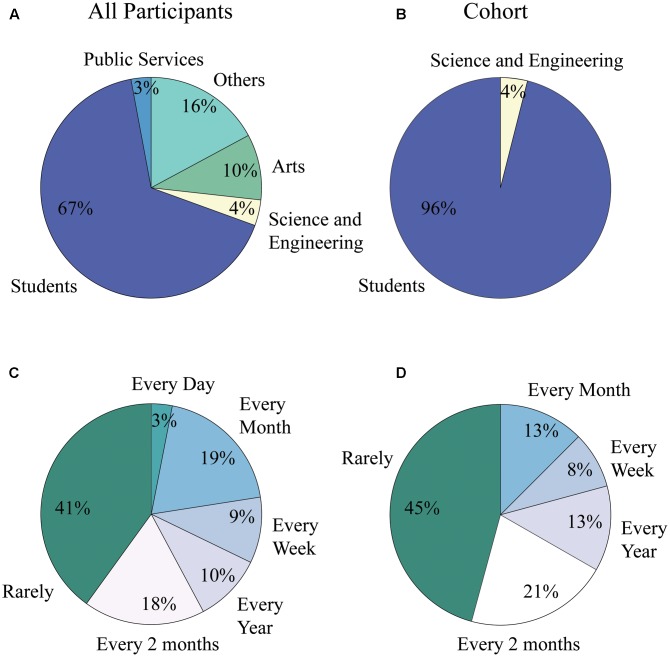
Art consumption levels and occupation distribution of population and cohort. **(A)** Occupational distribution for all participants of the study. 67% were students while only 10% were people working in the field of the arts. **(B)** Occupational distribution for analysis cohort. 86% of subjects were students, while none were involved in professional activities within the field of the arts. **(C)** Art consumption level for all participants of the study. Forty one percent were in contact with the arts less than one time every 2 months, while only 3% of them experienced art on a daily basis. **(D)** Art consumption level for analysis cohort. Forty five percent were in contact with the arts less than one time every 2 months, while none of the subjects experienced art on a daily basis.

Additionally, the majority of participants declared to have low art consumption levels, being exposed to art less than one time every 2 months (41% for all participants and 45% for analysis cohort); participants were also mainly students in both the general population and the analysis cohort (67% for all participants and 86% for cohort), (see **Figure 5**).

#### Artistic Preference Depended on Age

**FIGURE 6:**
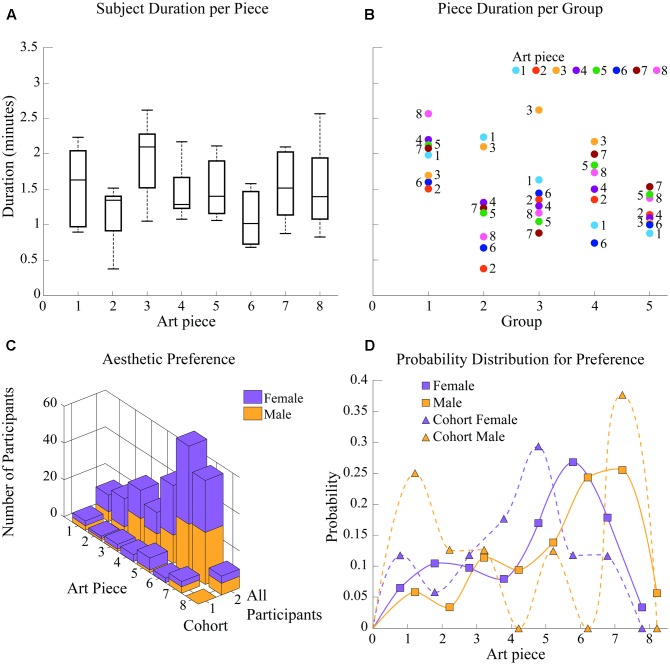
Group behavior and appreciation time. **(A)** Subject appreciation time per art piece. Art pieces 3 and 1 present the longest average appreciation times while art piece 8 shows the largest variation of appreciation durations among subjects. **(B)** Piece appreciation time per group. Group 6 shows the least variation in appreciation durations while group 1 spent significantly more time in each piece than any other group. Groups 3 and 4 spent significantly more time viewing art pieces 1 and 3. **(C)** Aesthetic preference per subject. For the general population, piece 6 “Saint Christopher IV” was the preferred one, while number 5 “Metropolis” was the overall favorite for the cohort. **(D)** Probability distribution for aesthetic preference. Preference shown in **(C)** is analyzed as a distribution of probability for accurate comparison of preference shifts between cohort and population.

With respect to their aesthetic preference, participants within the entire experimental population favored art pieces number 5, 6, and 7, with 7 being the overall favorite one (**Figure 6C**). These three pieces are similar in that they are the most realistic pieces of the eight that were included in the study. Pieces 6 and 7, which are the two top choices, approach the topics of religion and death as part of war; compared to piece 5, these two are even more brimming with a classical painting style focused on realism and detail. Piece 5 approaches the topic of social realities of poverty and decadence coexisting with opulence and entertainment. Looking into gender differences, females favored piece 6, religious art; males favored piece 7 “The War.” In the case of the analysis cohort, preferences were more diverse; nonetheless, piece 5 was clearly the cumulative favorite (**Figure 6C**).

As shown in **Figure 6D**, which shows probability distribution for favorite piece selection, female preference shifted from piece 6 “Saint Christopher IV” in the experimental population to piece 5 “Metropolis” in the cohort. Preference in the female cohort shows a difference in probability (probability of favorite piece being selected as favorite compared to the next most probable option) of pffc = 0.1176 (*p for favorite painting of female cohort)* while preference in the population shows a difference of pffp = 0.0894 (*p for favorite painting of female population)* < pffc, indicating females in the cohort favored piece 5 more than females in the general population favored piece 6. The difference in aesthetic preference distributions between these two groups (one, 6–88; the other, 18–30 years old) was significant at *p* = 0.0015).

Male aesthetic preference for the cohort remained on piece 7 “The War”; however, within the general population of participants (additionally including males over 30 years old), preference was almost equivalent for both piece 6 and piece 7 (topics: religion and death as part of war, respectively). This implies a significant shift (*p* = 0.0021) in aesthetic preference distribution for males between 18 and 30 years of age. Within the cohort, piece 6 showed a probability of being chosen as the favorite painting of p6mc = 0.00 (*p of selecting piece 6 for male cohort)*, compared to a probability of p6mp = 0.2442 (*p of selecting piece 6 for male population)* for the population; additionally, piece 7 was established as favorite in the male cohort with a difference in probability (probability of favorite piece being selected as favorite compared to the next most probable option) of pfmc = 0.125 compared to the difference in probability pfmp = 0.0116 for the favorite piece selected by males of the experimental population.

#### Behavior

Appreciation durations did not significantly vary between art pieces, staying within ∼1 and ∼2.2 min per piece, for most subjects and therefore most groups; however, art piece 3 had the greatest appreciation duration, with an average of ∼2 min, while art piece 8 had the largest variation (var = 0.4374 compared to the averaged variance of 0.2417 for all other pieces) of appreciation durations among subjects, presenting the most distributed durations from 0.83 to 2.57 min for that single art piece. These results are shown in **Figure 6A**. Note that appreciation durations were defined by guides, meaning they were independent of the aesthetic preference of subjects.

Differences in group behavior are also shown in **Figure 6B**. Although most groups varied in times per piece by approximately 1.5 min, group 5 showed the least variation (var = 0.049 compared to the averaged variance of 0.27 for all other groups and the second-smallest variance of 0.1243 for group 1) in appreciation durations, varying only 0.62 min from the least time spent of an art piece to the most time spent on another. Groups 2 and 3 spent the most time viewing art pieces 1 and 3 (2.23 and 2.1 min compared to the group’s average of 1.24 min per piece), and art piece 3 (2.62 min compared to the group’s average of 1.43 min per piece), respectively. Additionally, group 1 showed the largest durations per piece of all experimental groups (difference significant for groups 2, 3, and 5 at *p* = 0.0152, 0.0329, and 0.00011, respectively), starting on ∼1.5 min spent on art pieces 2 and 6 and going up to 2.57 min on art piece 8.

### Neural Activity Related to Aesthetic Preference in Young Adults

As described in Section “Demographics, Artistic Preference and Subject Behavior” and shown in **Figure 4**, a second, smaller subject cohort was selected from the participant pool to perform analyses of EEG signals while avoiding the age confound and thus age-dependent aesthetic preferences. Thus, this group was selected from the entire pool of participants in accordance, primarily, to age. To avoid the confound of age, the analysis cohort was comprised only of 18-30 year-olds (121 out of 209 participants or 57.89% were 18–30 year-olds). Participants within this analysis cohort were excluded if any of the following conditions were applicable: the subject’s records showed (1) inconsistencies in logging from one staff team to another, (2) gum chewing during the tasks, (3) excessive head motion, or (4) talking to other participants. After applying the above criteria, 96 (79.34%) participants in this age group were discarded. The final analysis sample (Nc = 25) consisted of 8 males and 17 females (20.66% of participants in the 18–30 year-olds group).

For these 25 subjects, cluster analysis was performed on 1,531 and 1,312 epochs for BL and FP data, respectively, with *Kn* correspondence related to 6 and 8 variables (male, female, and each of four sensors, in BL; male, female, each of four sensors, and presence/absence of explanation, in FP) in two different cluster sets (the first set with two clusters and one set with 7 clusters for BL; 9 clusters for FP).

Epochs within each independent cluster are identified in relation to the variables they represent. The percentage of epochs related to each of the 6 potentially separable variables with Kn correspondence is reported. This allows for evaluation of actual separability of EEG-signals in correlation with the 6 variables included in the study.

In BL data set *Kn2* = 7, while in FP, *Kn2* = 9 as “Explained” and “Not Explained” variables are nonexistent for data gathered as baseline. For both BL and FP data sets, Kn1 = 2 according to CH index analysis (Equations 1–5).

#### Neural Activity during Baseline Condition

Baseline data set was segmented into 2 and 7 clusters following each of the two different segmentation approaches; however, epoch correspondence for 2 clusters in BL resulted in *K1* having 320 exemplar epochs and K2 having 1211. This meant 79% of data was grouped into cluster 2, and 21% into cluster 1, which was not helpful in determining independence of variables. Correspondence for 7 clusters, however, was: K1: 67, K2: 253, K3: 153, K4: 250, K5: 182, K6: 191 and K7: 435 epochs. Clusters with the most epochs also showed to contain high percentages of all variables, and were therefore considered as containing noise epochs within each independent variable’s characteristic PSDs.

**FIGURE 7:**
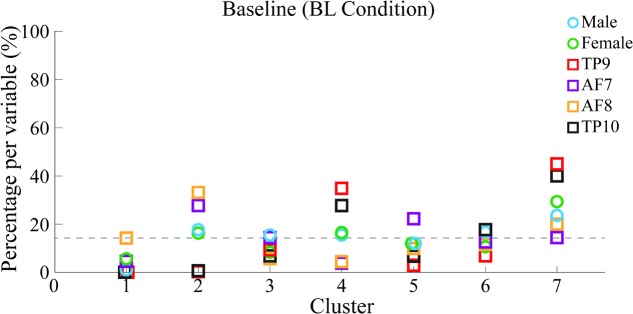
Percentages of epoch correspondence as a function of gender and channel location within each cluster for BL. Six different colors are used for 6 different variables to which epochs within each independent cluster are related. Percentages of epochs within each cluster related to each variable are shown. A horizontal line shows the average percentage of epoch-to-variable relation across clusters. 100% of epochs related to each variable are distributed between clusters, meaning a greater percentage of a specific variable within a specific cluster would indicate more epochs related to such variable were grouped into a single cluster. Percentages over this threshold are considered indicators of significant cluster-to-variable relation.

Percentages of epoch correspondence per variable within each cluster are shown in **Figure 7** along the vertical axis, while a horizontal line shows average correspondence of all epochs related to a single variable along clusters.

Results show, for BL data, frontal sensors AF7 and AF8 were mainly clustered into cluster 2 of 7 that were generated (cluster 2/7), indicating high similarity in PSDs. In the same way, temporo-parietal sensors TP9 and TP10 were clustered into cluster 4/7 and 7/7, also implying high similarity in their corresponding PSDs.

Cluster 7/7 contains the highest concentration of PSDs, potentially indicating low-signal-to-noise PSDs or common spectral patterns across variables. 7/7 is therefore not considered as the correspondence of the highest percentage of good-signal PSDs for any variable.

Additionally, the highest concentration of female PSDs (excluding epochs in K7, considered to be data with low signal-to-noise ratio) have a correspondence of cluster 2/7 and cluster 6/7, coinciding with that of frontal sensors AF7 and AF8 in cluster 2/7. The highest concentration of male PSDs (also excluding epochs in K7) have a correspondence of 2/7 and 5/7, also coinciding with that of frontal sensors in cluster 2/7; additionally, sensors AF7 and AF8 also have high correspondence with cluster 5/7.

**FIGURE 8:**
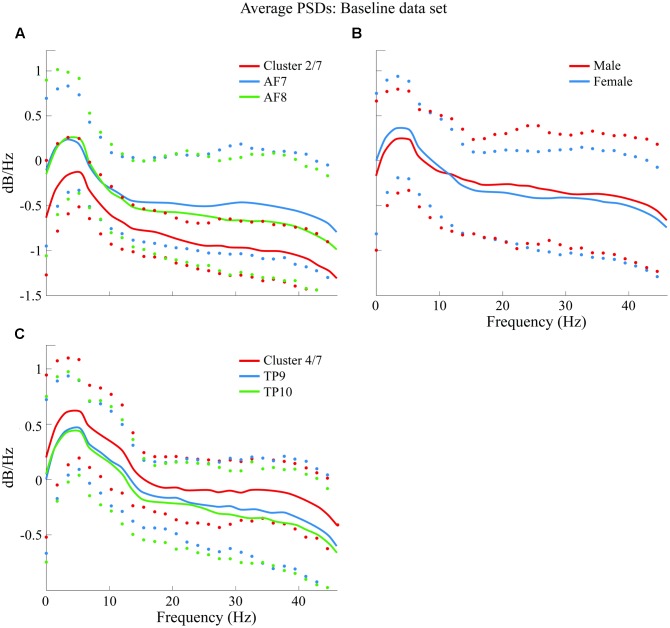
Comparison of average PSDs for specific clusters and variables in BL data set. **(A)** Cluster 2 of 7 clusters that were generated (*K2* of 7 or cluster 2/7) in BL data set compared to average PSDs of AF7 and AF8. **(B)** Comparison of male and female average PSDs, where no significant difference was found. **(C)** *K4* of 7 clusters in BL data set compared to average PSDs of TP9 and TP10. Confidence intervals for 90% of data are shown with markers above and below average curves; marker colors correspond with the curve they describe.

Average PSDs from clusters K2 and K4 are plotted and compared in **Figure 8** with average PSDs from all epochs related to variables AF7 and AF8 for K2; TP9 and TP10 for K4. Male and female average PSDs are also plotted and compared for BL.

#### Neural Activity during Perception of Favorite Painting

Favorite Painting data set was segmented into 2 and 9 clusters following each of the two different segmentation approaches. Epoch correspondence for 2 clusters in FP resulted as follows: *K1*: 845 and *K2*: 467 epochs; presenting the same situation as with BL for 2 clusters. The cluster size for the 9 clusters using the second segmentation approach was: *K1*: 162, K2: 84, K3: 100, K4: 270, K5: 229, K6: 155, K7: 74, K8: 141, K9: 97 epochs.

**FIGURE 9:**
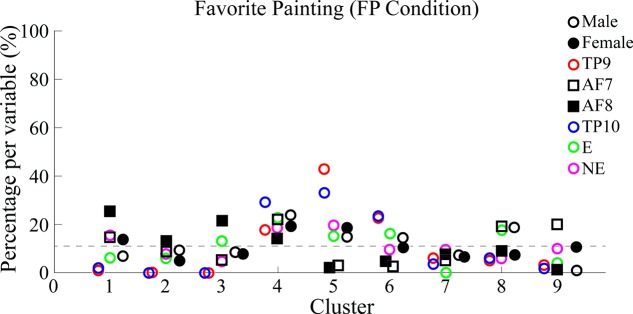
Percentages of epoch correspondence per variable within each cluster for FP. Figure characteristics are equivalent to those explained for **Figure 7**. Similarly to **Figure 7**, multiple clusters (in this case 1, 3, 5, 6, and 8) with a varying distribution of data points show both percentages under and above average. Specifically, cluster 5 best separated epochs from TP9 and TP10 from the rest. Again, the dotted line shows the average percentage of epoch-to-variable relation across clusters.

Percentages of epoch correspondence per variable within each cluster are shown in **Figure 9** along the vertical axis, while a horizontal line shows average correspondence of all epochs related to a single variable along clusters.

Results show, for FP data, temporo-parietal sensors were grouped into clusters 4/9 and 5/9 (indicate type of symbol used here) while frontal sensors were excluded from these clusters and scattered in clusters 2/9, 7/9, and 6/9, where they were grouped together in a similar way in which PSDs from frontal sensors AF7 and AF9 were grouped together in BL data set analysis.

Similarly to BL findings, male and female PSDs were grouped together. Interestingly, for FP data set analysis, male and female PSDs were grouped into clusters 4/9 and 5/9.

Additionally, highest concentrations of epochs relating to both “explained” and “not explained” variables were distributed among clusters without significantly grouping data in a specific one.

**FIGURE 10:**
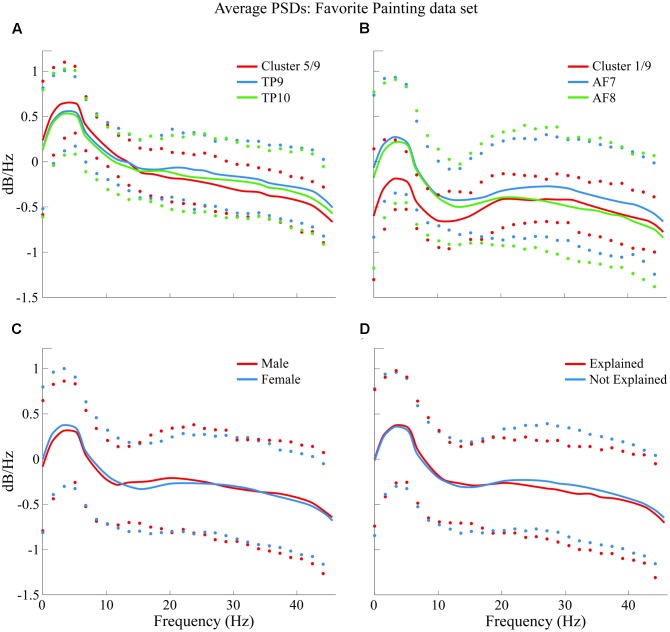
Comparison of average PSDs for specific clusters and variables in FP data set. **(A)** K5 of 9 clusters in FP data set compared to average PSDs of TP9 and TP10. **(B)** K1 of 9 clusters in FP data set compared to average PSDs of AF7 and AF8. **(C)** Male and female average PSDs for FP are compared. **(D)** “Explained” and “Not explained” average PSDs for FP are compared. Confidence intervals for 90% of data are shown with markers above and below average curves; marker colors correspond with the curve they describe.

Average PSDs from clusters *K5* and *K1* are plotted and compared in **Figure 10** with average PSDs from all epochs related to electrode variables TP9 and TP10 for *K5*; AF7 and AF8 for *K1*. Male and female average PSDs, as well as “Explained” and “Not explained” conditions, are also plotted and compared for FP.

#### Neural Activity Results on Baseline and Favorite Painting Comparison

**FIGURE 11:**
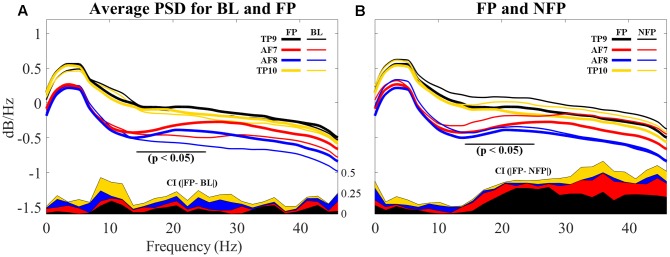
Comparison of average PSDs for all 4 individual sensors in BL and FP data sets. **(A)** Average PSDs for each sensor in BL data set compared to average PSDs for each sensor in FP. Individual FP sensor PSDs present suppression of frequencies visually evaluated to be between 12 and 20 Hz, partially coinciding with beta band (15–25 Hz) and particularly on frontal sensors AF7 and AF8. Additionally, alpha band (8–12 Hz) shows higher dB/Hz for BL compared to FP. **(B)** Average PSDs for each sensor in NFP data set compared to average PSDs for each sensor in FP. Smaller suppressions are noticeable for NFP PSD, for beta band. Differences also appear to be present in alpha band. On both **(A,B)**, differences between BL and FP **(A)** and FP and NFP **(B)** for confidence intervals (C.I.) on 90% of data are shown in the bottom of each graph; CI difference graphs are colored to correspond with the curves they describe. A black horizontal line shows where statistical difference was measured.

Finally, PSDs for all 4four sensors are individually compared for both BL and FP data sets. Epochs for all subjects were averaged for visualization and are shown in **Figure 11A**.

Additionally, resulting PSDs for each sensor were statistically compared using an independent two-sample *t*-test in order to evaluate if the suppression shown graphically proved to result in significantly different frequency characterizations of brain activities while viewing favorite paintings compared to baseline. Two different *p*-values were obtained for two different frequency segmentations: first, PSDs were statistically compared for each individual sensor considering all frequencies (1–50 Hz); second, PSDs were also compared specifically considering the beta frequency band (15–25 Hz). Average *p*-valu1es for the two frequency bands resulted as follows: for 1–50 Hz, *p* = 0.1558 (not significant); for beta band (15–25 Hz), pbeta = 0.0822 (significant at *p* < 0.10). Additionally, for the beta band p for AF7 = 0.0244 and p for AF8 = 0.0310, indicating significance at *p* < 0.05. Additionally, alpha band (8–12 Hz) was also evaluated for statistically significant difference; for this specific band, palpha = 0.0635 (also significant at *p* < 0.10), where p for TP9 = 0.0164 and p for TP10 = 0.0095, indicating significance at *p* < 0.05, for both temporo-parietal sensors.

In order to determine whether the measured beta suppression for FP compared to BL was related to the specific act of subjects viewing their favorite painting or to the general action of looking at a picture, one ancillary analysis was performed: for each subject, one non-favorite painting was selected at random for PSD analysis; the average PSD response across subjects was processed for epochs related to these randomly selected non-favorite paintings, in a process analogous to the analysis performed on BL and FP. Random selection was performed to provide a scenario that, after computing the average PSD for all non-favorite paintings, resembles the more general state of visual stimulation rather than stimulation with a specific aesthetic preference. The resulting average PSD response for this non-favorite state (NF) is shown in **Figure 11B**, compared to the average PSD response of FP. Statistical analysis through a two-sample *t*-test proved randomly selected non-favorite paintings produced a PSD response which was significantly different (*p* = 0.043) to that produced while subjects viewed their favorite paintings. In regard to alpha band, however, subjects’ PSD response between the general viewing state and the state of viewing their favorite painting did not show statistically significant difference.

## Discussion

### Age Influenced Preference of Art Style or Topic

In regard to demographics and behavioral analysis, subjects in the analyzed cohort were 18–30 years old with rare consumption of artistic content; museum newcomers who rarely experience art and who wish to approach it perhaps in a “simple” way by appreciating it in a place designed for it instead of looking for artistic stimuli in other alternative spaces requiring a greater effort investment. Guides in the museum dedicated approximately a minute or two to the appreciation of each art piece. The positive and negative effects of dedicating that specific amount of time to the consumption of art are arguable, and most artists will probably agree that more time should be dedicated to it. Interestingly, one guide dedicated from 4 to 6 min to each piece, perhaps influencing subjects in his group to repeat such behavior when appreciating art on their own. This group, however, had to be excluded from the analysis cohort due to such dissimilarity from the rest. Regardless of the small amount of time spent per piece, clear self-reported aesthetic preferences were measured for both the entire pool of participants in the study and for the smaller cohort of subjects. Additionally, statistically different preferences were found for this second group of subjects. This information may be taken into account by museums and the general artistic population, for different purposes. Within the entire experimental population realistic pieces with more classical styles of painting were favored; the topics of religion and death as part of war, as well as the topic of social realities of poverty and decadence coexisting with opulence and entertainment, were top favorite choices for the full study population. Females favored pieces of religious art, while males favored the topic of war. For the population between 18 and 30 years of age, females no longer preferred the religious topic and favored that of social reality; additionally, males no longer preferred both the topics of war and death as part of war as they now only favored the topic of war.

### Aesthetic Responses Were Associated with Beta Band Suppression in Prefrontal Cortices

With respect to the neuroaesthetic response of the participants, the main finding was that during aesthetic appreciation of favorite paintings (FP), subjects showed a significant suppression of the beta band (15–25 Hz) in electrodes AF7 and AF8, compared to the baseline condition (BL); furthermore, such suppression was not observed in the more general state of simple visual stimulation for the case of non-FPs. One previous study (Wagner et al., 2014) found similar beta band suppression related to the comparison of states perhaps different in their physical nature but similar in psychological terms: subjects’ neural activity was compared while performing a walking task in an engaged and in an unengaged manner. In their study, Wagner et al. (2014) found that engagement in the task, and sustaining what they called a “planning” mind state while performing it rather than simply following through, was correlated with such beta-band suppression. Their study, however, found beta suppression in premotor and parietal areas (in contrast to our findings of beta suppression over prefrontal electrodes); this may be related to the nature of the engaging task and it could be an indicator that beta-band suppression is more generally associated to engagement, whereas the spatial location of such suppression may be diversely dependent of the engaging task itself.

Interestingly, subjects in “Explained” and “Not Explained” groups in this study generated similar PSDs which were not grouped into independent clusters, indicating these variables were not separable for subjects between 18 and 30 years of age with the specific characteristics of being mostly students and having a low consumption of art. Following the aforementioned study, and considering that beta suppression in the frontal lobe was correlated with an increased engagement in a visual task, such similarity in brain activity regardless of whether or not subjects were provided with an explanation of art pieces could further imply that art viewers’ engagement is not determined by guided or un-guided viewing, but by whether or not they like what they see, regardless of what they are told about it.

An additional study (Griffiths et al., 2016) also found decreases in frequencies under 30 Hz (including beta band) when subjects were asked to memorize words associated to spatial locations along a path. It is therefore possible that an additional complementary explanation for the beta suppression found in our study be related to subjects’ interest in the painting they found to be their favorite. Subjects in this study may have attempted (perhaps as an unconscious response) to memorize the painting; a painting which, because of the experimental setup, may easily be associated to a spatial location within the museum and along a path which was being followed through the exhibit.

Finally, frontal and temporo-parietal sensors were grouped into mutually exclusive clusters in both BL and FP data set analysis. Such exclusiveness potentially indicates significant differences in beta suppression generated in these two brain areas in the prefrontal electrodes (the reader is referred to **Figure 11A**). Such beta suppression may be related to task engagement in relation to spatial memory for locations of preferred art because beta-suppression was not found in every piece, but only in FP.

As it can be noted in **Figures 11A,B**, sensors located over the temporo-parietal locations exhibited a positive shift in overall power as compared to sensors over the frontal lobe. This anterior-posterior cortical asymmetry resembles results shown by Hashemi et al. (2016), whose study evaluated differences on male and female subjects while performing a variety of tasks in an uncontrolled environment, using the same EEG headset used in this study.

With respect to alpha band, results showed a statistically significant decrease in PSD response in subjects’ state of viewing their favorite painting compared to baseline state; however, comparison with subjects’ general state of viewing showed no significant difference. This indicates the measured decrease in PSD response on FP vs BL could likely be associated only to the more general state of visual stimulation.

### Limitations and Future Work

Large amounts of data were deemed useless for analysis due to presence of artifacts as well as due to difficulty in accurate manual log-keeping for plentiful groups. The former seemed to be intensified in this study as a consequence of interaction between subjects during the experimentation process: head motion, talking, blinking were problematically common during recordings, as well by the fact that proper contact between electrodes and scalp was also easily compromised; the latter was, on the other hand, related to a limited number of staff members, which made it difficult for all groups to be properly accompanied by an appointed logger. Additionally, difficulties with videocamera placement on subjects, recordings were not good enough to assess information not already provided by the experimental log. It was not feasible to identify when subjects were looking at the art piece and when they were not. However, participants’ distractions were included in the written log.

Although “explained” and “not explained” variables (referring to presence and absence of explanation during appreciation of visual arts) did not show differences in brain activity generated for the specific cohort analyzed, subjects from a different age group and with different frequencies of art consumption should be studied to determine if such variables are truly not separable, or if they simply were not separable for this specific cohort.

Future MoBI studies would benefit from ensuring all time-related events are effectively logged so as to not lose data because of an impossibility of relating it to specific events. Although automating the process seems to be the first alternative that comes to mind, this presents development, implementation and reliability problems that were encountered in this study and that would also need to be addressed as part of the validation of the experiment; our recommendation is to ensure enough staff is included in the experimental protocol and create redundancy in manual logging for reliability purposes. Due to technical, usability, and museum constrains, it was not possible to record eye movements from the participants, which could have been used to know how long they actually looked at a certain painting. Additionally, ensuring brain signals are not visible to subjects prior to, or during, the experimental process could significantly aid participants’ concentration. We recommend showing real-time readings until the experiment is over. Finally, and in relation to the findings presented here, future MoBI studies could find interest in focusing attention on beta band response to different types of aesthetic stimuli. This study focused on favorable aesthetic experience vs. no aesthetic experience, specifically in visual art; studies could be conducted to analyze brain responses considering art pieces with varying grades of favourability by subjects and including other types of art such as music, literature or theater.

## Author Contributions

GH-A: algorithmic implementation, data analysis, interpretation strategies and manuscript preparation. JT-D: experimental design, data analysis, interpretation and validation strategies, statistical analysis, and final manuscript preparation. EA-D-A: data processing strategies. KK-L: data processing strategies. Md-A: experimental protocol implementation and data collection. UT-D: experimental protocol supervision. JC-V: technical and scientific supervision. RS: technical and scientific supervision. All authors edited and approved the final version of the manuscript.

## Conflict of Interest Statement

The authors declare that the research was conducted in the absence of any commercial or financial relationships that could be construed as a potential conflict of interest.

## References

[B1] Abdulkader S. N. Atia A. Mostafa-Sami M. (2015). Brain computer interfacing: applications and challenges. *Egypt. Inform. J.* 16 213–230. 10.1016/j.eij.2015.06.002

[B2] Aviv V. (2014). What does the brain tell us about abstract art? *Front. Hum. Neurosci.* 8:85. 10.3389/fnhum.2014.00085 24616683 PMC3937809

[B3] Beaty R. E. Benedek M. Silvia P. J. Schacter D. L. (2016). Creative cognition and brain network dynamics. *Trends Cogn. Sci.* 20 87–95. 10.1016/j.tics.2015.10.004 26553223 PMC4724474

[B4] Bono V. Das S. Jamal W. Maharatna K. (2016). Hybrid wavelet and EMD/ICA approach for artifact suppression in pervasive EEG. *J. Neurosci. Methods* 267 89–107. 10.1016/j.jneumeth.2016.04.006 27102040

[B5] Brieber D. Nadal M. Leder H. Rosenberg R. (2014). Art in time and space: context modulates the relation between art experience and viewing time. *PLOS ONE* 9:6. 10.1371/journal.pone.0099019 24892829 PMC4043844

[B6] Brown S. Gao X. Tisdelle L. Eickhoff S. B. Liotti M. (2011). Naturalizing aesthetics: brain areas for aesthetic appraisal across sensory modalities. *Neuroimage* 58 250–258. 10.1016/j.neuroimage.2011.06.012 21699987 PMC8005853

[B7] Cruz-Garza J. Kopteva A. Paek A. Contreras-Vidal J. L. (2016). *Your Brain on Art: Examining the Neural Substrate of Creativity in the Arts Using Mobile Brain-Body Imaging (MoBI).* San Diego, CA: Society for Neuroscience.

[B8] Fetz E. E. (2012). Artistic explorations of the brain. *Front. Hum. Neurosci.* 6:9. 10.3389/fnhum.2012.00009 22347178 PMC3273889

[B9] Griffiths B. Mazaheri A. Debener S. Hanslmayr S. (2016). Brain oscillations track the formation of episodic memories in the real world. *Neuroimage* 143 256–266. 10.1016/j.neuroimage.2016.09.021 27622395

[B10] Gwin J. T. Gramann K. Makeig S. Ferris D. P. (2010). Removal of movement artifact from high-density EEG recorded during walking and running. *J. Neurophysiol.* 103 3526–3534. 10.1152/jn.00105.2010 20410364 PMC3774587

[B11] Hashemi A. Pino L. Moffat G. Mathewson K. Aimone C. Bennett P. (2016). Characterizing population EEG dynamics throughout adulthood. *eNeuro* 3:ENEURO.0275-16.2016. 10.1523/ENEURO.0275-16.2016 27957533 PMC5150228

[B12] Huang M. (2009). The neuroscience of art. reviews and features. *Stanford J. Neurosci.* 2 24–26.

[B13] Ieracitano C. Duun-Henriksen J. Mammone N. La Foresta F. Morabito F. (2017). “Wavelet coherence-based clustering of EEG signals to estimate the brain connectivity in absence epileptic patients,” in *Proceedings of the International Joint Conference on Neural Networks (IJCNN)* (Anchorage, AK: IEEE). 10.1109/IJCNN.2017.7966002

[B14] Kontson K. L. Megjhani M. Brantley J. A. Cruz-Garza J. G. Nakagome S. Robleto D. (2015). Your brain on art: emergent cortical dynamics during aesthetic experiences. *Front. Hum. Neurosci.* 9:626. 10.3389/fnhum.2015.00626 26635579 PMC4649259

[B15] Leslie G. Ojeda A. Makeig S. (2014a). Measuring and classifying musical engagement using EEG and motion capture. *Psychol. Music* 39 18–49.

[B16] Leslie G. Ojeda A. Makeig S. (2014b). Measuring musical engagement using expressive movement and EEG brain dynamics. *Psychomusicol. Music Mind Brain* 24 75–91. 10.1037/pmu0000031

[B17] Liu Y. Sourina O. Nguyen M. K. (2011). Real-time EEG-based emotion recognition and its applications. *Trans. Comput. Sci.* 6670 256–277. 10.1007/978-3-642-22336-5_13 24312569

[B18] Makeig S. Gramann K. Jung T. P. Sejnowski T. J. Poizner H. (2009). Linking brain, mind and behavior. *Int. J. Psychophysiol.* 73 95–100. 10.1016/j.ijpsycho.2008.05.006 19414039 PMC2796545

[B19] Makeig S. Leslie G. Mullen T. Sarma D. Bigdely-Shamlo N. Kothe C. (2011). “Analyzing brain dynamics of affective engagement,” in *Lecture Notes in Computer Science*, Vol. 6975 (Berlin: Springer-Verlag).

[B20] Mastandrea S. Umiltà M. A. (2016). Futurist art: motion and aesthetics as a function of title. *Front. Hum. Neurosci.* 10:201. 10.3389/fnhum.2016.00201 27242471 PMC4868917

[B21] Ordikhani-Seyedlar M. Lebedev M. A. Sorensen H. B. D. Puthusserypady S. (2016). Neurofeedback therapy for enhancing visual attention: state-of-the-art and challenges. *Front. Neurosci.* 10:352. 10.3389/fnins.2016.00352 27536212 PMC4971093

[B22] Oude Bos D. (2006). *EEG-Based Emotion Recognition-The Influence of Visual and Auditory Stimuli.* Rotterdam: Capita Selecta.

[B23] Picard R. W. Vyzas E. Healey J. (2001). Toward machine emotional intelligence: analysis of affective physiological state. *IEEE Trans. Pattern Anal. Mach. Intell.* 23 1175–2001. 10.1109/34.954607

[B24] Rached T. S. Perkusich A. (2013). “Emotion recognition based on brain-computer interface systems,” in *Brain-Computer Interface Systems - Recent Progress and Future Prospects*, ed. Fazel-Rezai R. (Rijeka: InTech), 253–270.

[B25] Schaaff K. Schultz T. (2009). “Towards an EEG-based emotion recognizer for humanoid robots,” in *Proceedings of the 18th IEEE International Symposium on Robot and Human Interactive Communication* (Hoskote: Toyama), 792–796. 10.1109/ROMAN.2009.5326306

[B26] Shourie N. Firoozabadi M. Badie K. (2014). Analysis of EEG signals related to artists and nonartists during visual perception, mental imagery, and rest using approximate entropy. *BioMed Res. Int.* 2014:764382. 10.1155/2014/764382 25133180 PMC4123523

[B27] Speier W. Arnold C. Pouratian N. (2016). Integrating language models into classifiers for BCI communication: a review. *J. Neural Eng.* 13:031002. 10.1088/1741-2560/13/3/031002 27153565 PMC5495144

[B28] Vessel E. A. Starr G. G. Rubin N. (2013). Art reaches within: aesthetic experience, the self and the default mode network. *Front. Neurosci.* 7:258. 10.3389/fnins.2013.00258 24415994 PMC3874727

[B29] Wagner J. Solis-Escalante T. Grieshofer P. Neuper C. Müller-Putz G. Scherer R. (2012). Level of participation in robotic-assisted treadmill walking modulates midline sensorimotor EEG rhythms in able-bodied subjects. *Neuroimage* 63 1203–1211 10.1016/j.neuroimage.2012.08.019 22906791

[B30] Wagner J. Solis-Escalante T. Scherer R. Neuper C. Müller-Putz G. (2014). It’s how you get there: walking down a virtual alley activates premotor and parietal areas. *Front. Hum. Neurosci.* 8:93. 10.3389/fnhum.2014.00093 24611043 PMC3933811

[B31] Yu M. Gouw A. Hillebrand A. Tijms B. Jan Stam C. van Straaten E. (2016). Different functional connectivity variant of frontotemporal dementia and Alzheimer’s disease: an EEG study. *Neurobiol. Aging* 42 150–162. 10.1016/j.neurobiolaging.2016.03.018 27143432

